# Applications of Ruthenium Complex in Tumor Diagnosis and Therapy

**DOI:** 10.3389/fphar.2018.01323

**Published:** 2018-11-19

**Authors:** Ke Lin, Zi-Zhuo Zhao, Hua-Ben Bo, Xiao-Juan Hao, Jin-Quan Wang

**Affiliations:** ^1^School of Bioscience and Biopharmaceutics, Guangdong Province Key Laboratory for Biotechnology Drug Candidates, Guangdong Pharmaceutical University, Guangzhou, China; ^2^Department of Ultrasound, Sun Yat-sen Memorial Hospital, Sun Yat-sen University, Guangzhou, China; ^3^Manufacturing, Commonwealth Scientific and Industrial Research Organisation, Clayton, VIC, Australia

**Keywords:** ruthenium complexes, antitumor, diagnosis and therapy, drug combinations, synergistic effect

## Abstract

Ruthenium complexes are a new generation of metal antitumor drugs that are currently of great interest in multidisciplinary research. In this review article, we introduce the applications of ruthenium complexes in the diagnosis and therapy of tumors. We focus on the actions of ruthenium complexes on DNA, mitochondria, and endoplasmic reticulum of cells, as well as signaling pathways that induce tumor cell apoptosis, autophagy, and inhibition of angiogenesis. Furthermore, we highlight the use of ruthenium complexes as specific tumor cell probes to dynamically monitor the active biological component of the microenvironment and as excellent photosensitizer, catalyst, and bioimaging agents for phototherapies that significantly enhance the diagnosis and therapeutic effect on tumors. Finally, the combinational use of ruthenium complexes with existing clinical antitumor drugs to synergistically treat tumors is discussed.

## Introduction

**FIGURE 1:**
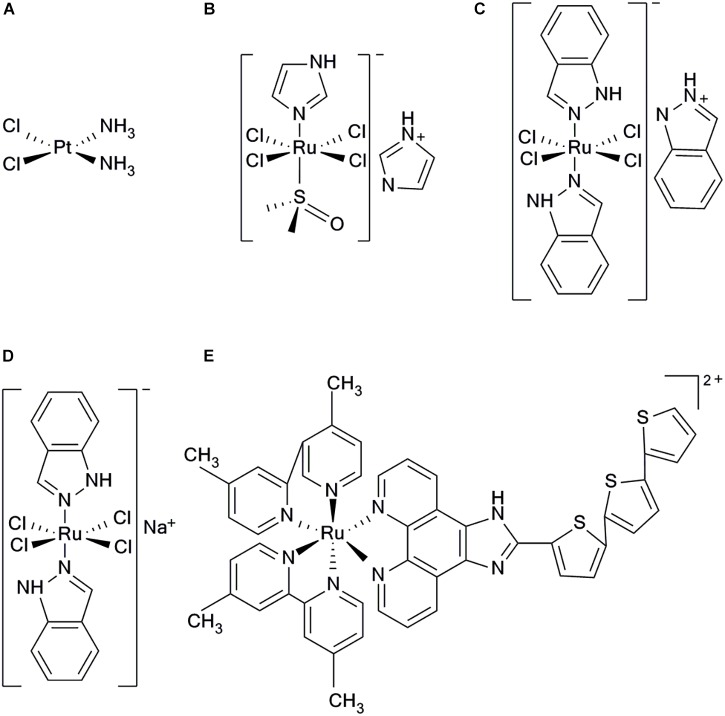
Structure of five clinical complexes; **(A)** Cisplatinum, **(B)** NAMI-A, **(C)** KP1019, **(D)** KP1339, and **(E)** TLD1443.

Chemotherapy is an important modality for cancer treatment. Since the introduction of metal chemotherapeutics represented by cisplatinum (Figure 1A), numerous metal agents have been developed as antitumor drugs, and platinum-based drugs have become the focus of metal-based antitumor drug research (Harper et al., 2010; Burger et al., 2011; Wang X. et al., 2015). In recent years, the platinum-based drugs have become the first line of anti-cancer drugs because of their significant antitumor efficacy (Jakupec et al., 2008; Gasser et al., 2011; Wang and Guo, 2013). However, there are increasing reports that platinum-based anticancer drugs have severe side effects including myelotoxicity, peripheral neuropathy et al. (Galanski, 2006; Samimi et al., 2007). Therefore, researchers have turned their attention to other potential metal antitumor drugs. Ruthenium complexes have shown remarkable antitumor activity among the numerous metal compounds studied; they possess various advantages over platinum drugs, such as potent efficacy, low toxicity, less drug resistance, and are expected to become a new generation of clinical metal antitumor drugs (Abid et al., 2016; Thota, 2016; Southam et al., 2017).

There are three main oxidation states of ruthenium compounds. The high oxidation state of Ru(IV) compound is unstable, which limited its further development (Duan et al., 2009). Ru(III) complexes have good stability of thermodynamics and kinetics, and can be used as prodrugs under biological circumstances of hypoxia, acidic pH and high level glutathione, showing antitumor effect by reducing to corresponding Ru(II) counterparts *in vivo* (Minchinton and Tannock, 2006; Antonarakis and Emadi, 2010). Ru(II) can directly kill tumor cells *via* multiple mechanisms (Zeng et al., 2015). Ru(II) complexes have great photophysical and chemical properties as well as multiple exchanging ligands. Combining with their applicability as nanomaterials and they have demonstrated significant antitumor efficacy (Poynton et al., 2017). Generally, the thermodynamic and kinetic stability of Ru(II) compounds are higher than Ru(III) due to their lower oxidation states (Duan et al., 2009). In addition, the nature and net charge of the ligands play important roles in the kinetics of Ru(II) compounds hydration (Abid et al., 2016). Many Ru(II) compounds showed better antitumor activities than their corresponding Ru(III) counterpart *in vivo* (Minchinton and Tannock, 2006; Hartinger et al., 2013). Generally speaking, the following options are viable in improving the water solubility of ruthenium compounds. (i) modifying the ligand structures; (ii) constructing the supramolecular ruthenium compounds; (iii) encapsulating ruthenium compounds into nanomaterial systems. (Suss-Fink, 2010; Jiang et al., 2012; Schmitt et al., 2012).

All the following ruthenium complexes that have progressed to clinical studies, NAMI-A {ImH[*trans-*RuCl_4_(dmso) (imidazole)]} (Figure 1B), KP1019 {indazolium *trans-*[tetrachlorobis(1H-indazole)ruthenate(III)]} (Figure 1C), and KP1339, are Ru(III) complexes (Webb et al., 2013). NAMI-A showed potent inhibitory efficacy on tumor metastasis. However, the phase II clinical studies revealed that it caused severe side effects in patients and, therefore, further investigations were not undertaken (Bergamo et al., 2003; Alessio et al., 2004). KP1019 had also failed to be investigated because of its poor water solubility, severe side effects and unsatisfactory efficacy for clinical study, (Hartinger et al., 2006, 2008). To improve the low water solubility of KP1019, researchers designed a more soluble sodium salt complex, KP1339 [Na(*trans-*RuCl_4_ (Ind)_2_)] (Figure 1D), which is currently used in clinical studies (Heffeter et al., 2010). Using the potent photophysical and chemical properties of Ru(II) complex, researchers have synthesized a photosensitizer TLD1443 (Figure 1E), which has immensely enhanced photodynamic therapy (Zeng et al., 2017a). It has a significant therapeutic efficacy on bladder cancer and is currently in phase II clinical trials (Smithen et al., 2017).

Based on the characteristics of ruthenium compound, optimizing its structure with relevant modification is a good strategy to improve its targeting capability and antitumor activity (Blanck et al., 2012). Researchers designed a series of lipophilic ruthenium complexes that effectively increase the uptake efficiency of tumor cells (Svensson et al., 2010; Matson et al., 2011). They found that the difference in the length of alkyl ether chains contributed to the different organelle-targeting properties of ruthenium complexes. Coupling of targeted polypeptides with ruthenium complexes is another effective way to enhance their targeting capability (Chakrabortty et al., 2017). In addition, encapsulating ruthenium complexes into nanomaterials can improve their targeting capability through the enhanced permeation and retention (EPR) effect (Frasconi et al., 2013; Wei et al., 2015). Capitalizing the properties of Ru(II) complexes, researchers have designed a series of nanoruthenium complexes including, Ru(II)-selenium nanoparticles (Sun et al., 2013), Ru(II)-gold nanocomplexes (Rogers et al., 2014), Ru(II)-silicon nanocomplexes (Frasconi et al., 2013), Ru(II)-carbon nanotubes (Wang N. et al., 2015), and some organic and biometallic nanoruthenium complexes (Chakrabortty et al., 2017) with direct antitumor effects. These nanoruthenium complexes can also be used as a good catalyst, photosensitizer and tracer to enhance the therapeutic effect (Chakrabortty et al., 2017).

## Antitumor Targets and Mechanisms of Ruthenium Complexes

**FIGURE 2:**
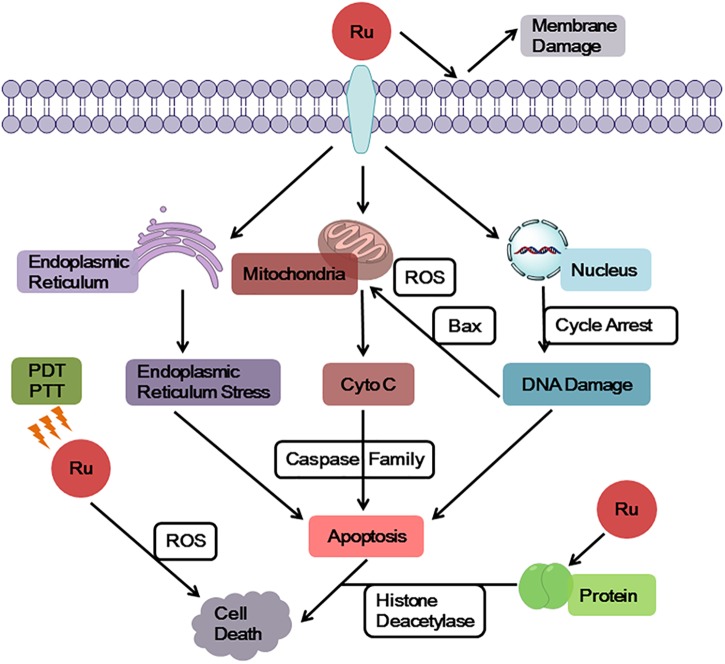
General targets and mechanisms of anticancer action of ruthenium complexes.

Ruthenium complexes show multiple targets and diverse mechanisms for its antitumor properties (Figure 2). Some ruthenium complexes act on telomere DNA, some interfere with replication and transcription of DNA, and others inhibit related enzymes (Kurzwernhart et al., 2012; Jain et al., 2018). Furthermore, ruthenium complexes can block the cell cycle (Kou et al., 2012; Wang et al., 2016; De Carvalho et al., 2018) and induce the formation of DNA photocrosslinking products to prevent RNA polymerization enzymes or exonucleases from binding to DNA, thereby causing tumor cell apoptosis (Le Gac et al., 2009; Rickling et al., 2010). Studies have found that some dinuclear and polynuclear Ru(II) polypyridyl complexes bind stably to the G-quadruplex (G4-DNA) structure of telomere DNA (Hiyama et al., 1995; Ambrus et al., 2006), inhibiting telomerase activity and blocking the function of DNA replication, thus, preventing normal cells from developing into immortalized tumor cells (Rajput et al., 2006; Shi et al., 2008). Ruthenium complexes have good topoisomerase (Topo) inhibitory activity (Kurzwernhart et al., 2012); however, some studies have found that inhibition of one type of Topo increases the activity of others (Crump et al., 1999; Vey et al., 1999). To solve this problem, studies have been conducted to synthesize a ruthenium complex with dual inhibitory property on Topo I and Topo II, which significantly inhibits tumor cell proliferation (Du et al., 2011; Zhang et al., 2013). Researchers have also designed a ruthenium complex with dual inhibitory effects on G4-DNA and Topo (Liao et al., 2015), achieving multitarget synergy with strong apoptosis promoting effects on tumor cells. In addition, Hurley and co-workers reported a ruthenium complex with dual stabilizing effects on Topo and G4-DNA, which also inhibited some drug resistant tumor cells (Kim et al., 2003).

In addition, it was found that ruthenium complexes accumulate more in organelles, such as mitochondria, endoplasmic reticulum, and lysosome, than in nucleus (Puckett and Barton, 2007; Groessl et al., 2011). A number of studies have revealed that mitochondria is a key target of ruthenium complexes (Wang et al., 2014; Liu et al., 2015; Wan et al., 2017), because ruthenium complexes can quickly decrease the membrane potential of mitochondria, leading to mitochondrial dysfunction or activating mitochondrial apoptosis pathways. Furthermore, this effect promoted the expression of pro-apoptotic members of the B-cell lymphoma-2 (Bcl-2) family, releasing cytochrome c (Cyto C), and activating cascade reactions of the caspase family members to induce tumor cell apoptosis. The endoplasmic reticulum is a key participant in tumor cell apoptosis, autophagy, and drug resistance and, thus, is a target in antitumor research (Sano et al., 2012; Fernandez et al., 2015). Ruthenium complexes can target the endoplasmic reticulum, cause oxidative stress or endoplasmic reticulum stress (ERS), and induce tumor cell apoptosis by activating caspase family members (Gill et al., 2013; Sano and Reed, 2013). In addition, ruthenium complexes can target another significant participant in autophagy, the lysosomes, inducing autolysosome production and hydrolase release (Tan et al., 2010; Castonguay et al., 2012; Chen et al., 2016). Thereby, they increase apoptosis of tumor cells (Yuan et al., 2015).

A very important feature of ruthenium complexes is that it is effective against many platinum resistant tumors. Gasser et al. found that [Ru(dppz)_2_(CppH)]^2+^ (CppH = 2-(20-pyridyl)-pyrimidine-4-carboxylic acid)] accumulated in the mitochondria. Moreover, this Ru(II) complex showed more cytotoxic effect in cisplatin-resistant A2780/CP70 cells than cisplatin and less cytotoxic than cisplatin in normal MRC-5 cells (Pierroz et al., 2012). Dyson and co-workers also designed some ruthenium complexes which contained ethacrynic acid (EA) ligands that inhibited cisplatin resistant A2780cisR cells (Ang et al., 2007). Moreover, Chao’s and Chen’s group designed a series of mitochondria-targeted Ru(II) complexes, based on a 2-phenylimidazo[4,5-f] [1,10]phenanthroline (PIP) Ru(II) polypyridyl complexes. These complexes induced apoptosis *via* a mitochondrial pathway and were effective against cisplatin resistant tumor cells (Li et al., 2012c; Wang et al., 2014; Yu et al., 2014).

The membrane structure as a “protective barrier” not only regulates the entry of drug molecules into cells, but also acts as a direct target of drug molecules, effectively killing tumor cells. A number of studies have confirmed that ruthenium complexes directly act on cell membrane, changing its permeability to allow cellular content to flow out of cells and induce cell apoptosis (Deng et al., 2017). Using the photophysical properties of Ru(II) complexes, researchers designed a Ru(II) polypyridine complex that accumulates on mitochondrial membrane and tumor surface membrane. These complexes emit red phosphorescence and produce a large amount of ^1^O_2_, thereby causing cytotoxicity and inducing cell apoptosis (Hess et al., 2017; Pal et al., 2018). Chao and colleagues synthesized Ru(II) pyridine complexes with two-photon performance and ^1^O_2_ yield, which could serve as a photosensitizer to simultaneously target surface membrane and mitochondrial membrane of human cervical carcinoma (HeLa) cells, achieving a dual killing effect (Qiu et al., 2017).

## The Use of Ruthenium Complexes in Diagnosis and Treatment of Tumors

The effective diagnosis and treatment of tumors is a major clinical challenge. Ruthenium complexes have shown promising application prospects to this difficulty. The combination of development and applications of subcellular targeting probes and bio-imaging technologies with the understanding of the occurrence and physiological development of tumors, is expected to facilitate the achievement of tumor-specific diagnosis and therapy. Ru(II) complexes have the advantages of considerable photothermal stability, large stokes shift, long luminescence lifetime, and low toxicity (Gill et al., 2009). They are ideal photosensitizers, catalysts, and imaging agents in phototherapy, and could serve as excellent probes and tracers for subcellular structure localization. Thomas and colleagues reported a lipophilic Ru(II) complex that can be used as a fluorescent probe, targeting the mitochondria and endoplasmic reticulum of human breast cancer cell (MCF-7), and it showed comparable cytotoxicity to that of cisplatin (Gill et al., 2013). In addition to targeting and imaging tumor subcellular structures, ruthenium complexes can also detect and specifically recognize biological components of the microenvironment. As a significant active ingredient in organisms, the level of thiol in tumor tissues can change rapidly. Specific recognition of the thiol level is important for tumor diagnosis and therapy (Dirican et al., 2016; Inal et al., 2017). The Ru(II)-gold nanocomplex synthesized by Chao and co-workers could be used as a specific two-photon probe for thiol level, as it detected biothiol levels in living HeLa cells and mouse hippocampus using two-photon microscopy, which provides a potent tool for molecular biology research in tumors (Zhang et al., 2014). The oxygen allotrope O_2_ is an indispensable source of metabolic energy and could be specifically identified and used to monitor the local metabolites of tumor cells, which would facilitate tumor diagnosis and therapy. Keyes and colleagues found that a peptide-bridged dinuclear Ru(II) complex as the mitochondrial fluorescent probe can monitor the dynamic changes of O_2_ concentration in mitochondria of HeLa cell, which could be used to monitor the malignant proliferation of tumor cells (Martin et al., 2014). The non-oxygen-dependent Ru(II) complex has been used as a photosensitizer in treating hypoxic tumors. This complex overcomes the limitations of low-depth-effect and low cell killing efficiency of phototherapy, significantly increasing ^1^O_2_ production and fluorescence efficiency, thus, enhancing cytotoxicity of ruthenium complex and showing potent therapeutic effects (Volker et al., 2014; Sadhu et al., 2015; Cuello-Garibo et al., 2017).

The development of DNA structure recognition and imaging probes enables us to understand the pathogenesis of cancer at the genetic level, which has enhanced the study of antitumor drugs. Using the optical switch effect of Ru(II) complex to DNA (Augustyn et al., 2007), a Ru(II) polypyridine complex as a DNA secondary structure recognition probe was designed. The Barton research team reported a selective Ru(II) complex for DNA mismatch detection and fluorescence localization, which effectively reduces the risk of carcinogenesis caused by base mismatches (McConnell et al., 2012). DNA bulge structures are caused by the DNA recombination process, which is likely to cause a frameshift mutation in DNA replication. This structure binds more tightly to DNA repair proteins than it does to normal double-stranded DNA, making the bulge structures a potential binding site for therapeutic drugs (Pieniazek et al., 2011). Keene and colleagues synthesized a series of binuclear Ru(II) complexes that selectively recognize and bind to DNA bulge structures *via* electrostatic interaction and zonal action, and have DNA-targeted repair function (Mulyana et al., 2011; Li et al., 2012a). Z-DNA induces gene deletion, translocation, and other instability (Dumat et al., 2016). Tridentate complexes, [Ru(tpy)(ptn)]^2+^ and [Ru(dmtpy)(ptn)]^2+^, were designed to induce Z-DNA transforms into a stable B-DNA dominant conformation, which effectively decreased the risk of mutations (Li et al., 2012b).

In addition to DNA imaging, some complexes were synthesized by coupling fluorescent Ru(II) complexes with histone deacetylase inhibitors (HDACIs). These complexes specifically recognize and image proteins (Kurzwernhart et al., 2012). Further investigation has found that it not only images and inhibits HDACs, but also produces a large amount of reactive oxygen species (ROS) under light irradiation, showing comparable cytotoxicity to that of cisplatin. Thus, it induces apoptosis of some tumor cells. Photoacoustic imaging (PA) is a novel imaging technique for tissue imaging based on optical absorption coefficients under the action of an imaging agent (Levi et al., 2014). Liu and co-workers used poly(nisopropylacrylamide) as a thermal response switch and [Ru(bpy)_2_(tip)]^2+^ as a photosensitizer in combination with gold nanomaterials to synthesize the Ru(II) complex pRu-pNIPAM@RBT (Chen et al., 2017). Under optical stimulation, this complex produces high heat and large amounts of ROS in tumor tissues, and it showed synergistic action in photothermal therapy (PTT) and photodynamic therapy (PDT) against tumors. Ruthenium complexes are good imaging agents for PA. Combination of infrared thermal imaging quantitative analysis and PA data, can be effectively used to distinguish healthy and tumor tissues, which has significantly improved the accuracy and efficiency of tumor therapy (Su et al., 2010).

At the organizational level, tumor cell proliferation and metastasis depend on adequate nutrient supply and angiogenesis. Therefore, blocking tumor angiogenesis is also a key strategy to inhibit tumor growth and migration (Gau et al., 2017). Studies have found that some ruthenium complexes have good antiangiogenic effects and effectively inhibit tumor growth (Silva Sousa et al., 2016). Liu and colleagues designed a fluorescent Ru(II)-selenium nanoparticles (Ru-SeNPs) that significantly inhibited the proliferation of liver carcinoma HepG2 cells. *In vivo* experiments in tumor bearing mice revealed that NAMI-A potently inhibited tumor angiogenesis and migration (Vacca et al., 2002). In another study, the nitric oxide synthase (NOS) pathway was found to play an important role in tumor angiogenesis (Chakraborty and Ain, 2017). Increasing NO levels is positively correlated with tumor growth and migration. Drugs that interfere with the NOS pathway can inhibit tumor angiogenesis. It has been observed that NAMI-A inhibits vascular endothelial growth factor (VEGF)-mediated angiogenesis in tumor tissues by scavenging NO (Morbidelli et al., 2003).

## Synergistic Effect of Ruthenium Complexes

Drug combinations are common therapeutic strategies in clinical practices. Combinational drug molecules act on multiple targets and pathways simultaneously, which could enhance their synergistic effects, reduce dosage and side effects, and reduce the risk of drug resistance (Lehar et al., 2009). A ruthenium complex was combined with a second-line antitumor agent ketoconazole (KTZ) in hormone-refractory cancer therapy to form a RuCl_2_(KTZ)_2_ complex, which showed a favorable synergistic effect (Bozic et al., 2013). The combination of these two agents in a C8161 melanoma cell line significantly enhanced the expression of caspase-3 and promoted tumor cell apoptosis. Mechanistic studies have shown that RuCl_2_(KTZ)_2_ has mitochondrial targeting effects, releasing mitochondrial cytochrome c and activating superoxide dismutase (Mn-SOD), thereby facilitating apoptosis. In the melanoma (WM164) cell line, RuCl_2_(KTZ)_2_ displayed a stronger inhibitory effect on tumor cell growth than cisplatin, and induced apoptosis by activating poly-ADP ribose polymerase (PARP) fragmentation and the proapoptotic factor Bcl-2-associated X protein (Bax) expression. RuCl_2_(KTZ)_2_ acts on the P53 signaling pathway to effectively inhibit the proliferation of a variety of adherent tumor cells, and synergizes the anti-epidermal growth factor receptor (EGFR) inhibitor C225MAb to kill resistant spheroids (Gelfo et al., 2016).

Berger and colleagues studied the combinations of ruthenium complexes and first-line anticancer drugs. They found that the clinical drug, ruthenium complex KP1339 combined with multi-kinase inhibitor sorafenib was more effective in the therapy of hepatoma (Hep3B) than KP1339 or sorafenib alone (Heffeter et al., 2013). Specifically, the mean survival of patients was extended by 3.9-fold by the combination, whereas KP1339 and sorafenib alone extended it by 2.4-and 1.9-fold, respectively. The combination of both agents effectively inhibited sorafenib-resistant tumor cells. In-depth investigations have found that the combination substantially increased their intracellular accumulation and, thereby, interfered with the DNA synthesis process, rendering the cells unable to perform effective mitosis, and enhancing apoptosis induction.

In clinical studies, NAMI-A combined with gemcitabine, better inhibited the activity of non-small cell lung cancer cells and reduced tolerance compared with the use of gemcitabine alone, but the combination of both had significant side effects such as neutropenia, anemia, and renal impairment (Leijen et al., 2015). Sava and co-workers identified promising drug combinations with synergistic potential using high-throughput screening (Bergamo et al., 2015). NAMI-A and doxorubicin were shown to have a potent synergistic antitumor efficacy. NAMI-A effectively increased the accumulation of doxorubicin in breast carcinoma. In *in vivo* studies of mouse MCa mammary carcinoma, this combination increased inhibition of tumor metastasis by 70%, compared to the use of doxorubicin alone. In a lung metastasis preclinical tumor model in mice, both agents demonstrated promising synergistic effects (Marien et al., 2017). However, there were noticeable side effects when the maximum doses were used.

The tumor vasculature is poorly organized, resulting in extravascular permeation of drug molecules (Pries et al., 2010). In addition, the decreased blood flow and oxygen supply affects drug uptake, which is also a major obstacle to effective tumor therapy (Siemann and Horsman, 2015). Studies on the combination of ovarian carcinoma chemotherapeutic doxorubicin and a ruthenium complex RAPTA-C have demonstrated that this combination significantly promoted the apoptosis of A2780 ovarian carcinoma cells compared with either single drug alone (Weiss et al., 2015). Normalization of tumor vasculature induced by apoptosis reduces vascular extravasation, and provides adequate oxygen for oxygen-dependent phototherapy, achieving synergism (Goel et al., 2011). These studies provide valid evidence for the interaction between anti-angiogenesis and antitumor effects.

## Conclusion and Perspectives

Investigation of the antitumor activity of ruthenium complexes has led to gratifying achievements and the identification of some promising antitumor compounds (Chen et al., 2016; Alves de Souza et al., 2017; Zeng et al., 2017b; Zhao et al., 2018). The ruthenium complex showed more potent activities than platinum drugs, and has a significant inhibitory effect on platinum-resistant tumor (Zeng et al., 2016). The peculiarity of ruthenium compounds suggests that the research methods used for investigating platinum-based drugs may not fully be applied in these agents, because the cytotoxic mechanisms of cisplatin and ruthenium are different. The primary target of cisplatin is DNA, but the target of some ruthenium complexes is mitochondria or endoplasmic reticulum. Although they can both regulate cell apoptosis and cell cycle, cisplatin induces a large number of genes related to DNA damage, P53 and apoptosis, while some ruthenium complexes facilitate the expression of oxidative stress and ER stress (Licona et al., 2017).

The existing research achievements should be combined with molecular biology and nanomaterials, applying the advantage of existing tools and methods to develop antitumor drugs with better therapeutic effects, based on these complexes. This prospect is extremely enlightening, and antitumor drugs with better efficacy than that of existing chemotherapeutic drugs, which are ineffective in treating certain tumors, could be developed. Furthermore, the prospective agents could be effective against tumors that have developed drug resistance for their potent efficacy (Wang N. et al., 2015; Purushothaman et al., 2018). The results of clinical studies should be reflectively considered in determining the reasons for the failure of the clinical investigations of NAMI-A and KP1019, which could lead to design drugs with less side effects, greater selectivity, and higher bioavailability. For example, KP1339, the sodium salt of KP1019, which is currently in clinical studies, has better water solubility and transmembrane absorption efficiency than KP1019 (Bytzek et al., 2016). The Ru(II) complex TLD1443, as a promising photosensitizer, significantly enhanced the efficacy of phototherapy and produced less toxicity *in vitro* and *in vivo* (Smithen et al., 2017).

Numerous breakthroughs have been made in the diagnosis and therapy of tumors using ruthenium complexes (Thota et al., 2018). As a probe, the ruthenium complex could be used for target localization and imaging of DNA, the mitochondria, endoplasmic reticulum, and lysosomes, achieving specific identification and dynamic monitoring of thiol and O_2_ in tumors (Martin et al., 2014; Zhang et al., 2014). As a tracer, it enhances the understanding of the physiological development of tumors at the genetic level (Wilson et al., 2016; Xu et al., 2016). As photosensitizers and catalysts, these complexes have significant synergistic effects with phototherapies such as PDT, PTT, and photoactivated chemotherapy (PACT) (Chen et al., 2017). The combination of ruthenium complexes and PA imaging technology has significantly improved the accuracy and effectiveness of tumor diagnosis and therapy (Chen et al., 2014). In the therapy of tumors using drug combinations, ruthenium complexes have shown favorable efficacy. The Ru(II) complex combined with KTZ significantly inhibited the proliferation of C8161 melanoma cells and directly killed cisplatin-resistant spheroids (Bozic et al., 2013). KP1339 combined with the first-line anticancer drug sorafenib for hepatic carcinoma, demonstrated a remarkable therapeutic effect (Heffeter et al., 2013). Furthermore, NAMI-A combined with gemcitabine enhanced the inhibitory effect on non-small cell lung cancer while NAMI-A combined with doxorubicin showed potent inhibitory effects on lung metastasis *in vivo* (Bergamo et al., 2015). RAPTA-C and doxorubicin showed synergistically enhanced therapeutic effects on ovarian cancer and some solid tumors (Weiss et al., 2015). However, studies on the synergistic effect of ruthenium complexes are rare, because there are some uncertain factors such as the mechanism of drug synergy and how to choose drugs that cooperate with ruthenium complexes (Zhao et al., 2013; Madani Tonekaboni et al., 2018).

In conclusion, the results of the investigations on drugs combinations with ruthenium complexes are currently unsatisfactory. Perhaps the development and use of high-throughput screening technology and algorithm analysis tools are a viable strategy to promote the study of drug synergistic effects (Aviolat et al., 2018).

Presently, the mechanism of action of ruthenium complexes is unclear, and further research is still needed. Before the ruthenium complex can be used clinically, numerous problems need to be addressed, including strategies to improve the hydrolysis of ruthenium complexes to achieve effective absorption and better metabolism, as well as enhance their cellular penetration to achieve targeted tumor cell death. Furthermore, methods to avoid and alleviate the side effects of ruthenium complexes, enhance their efficacy *via* synergism, and overcome drug resistance are imperative. The solution to these problems would provide a promising direction for the design and screening of ruthenium complexes, which are of great significance for their use in clinical diagnosis and therapy of tumors.

## Author Contributions

KL and Z-ZZ drafted and wrote the manuscript. J-QW conceived the idea for the manuscript. H-BB and X-JH provided critical analysis and language editing. All authors contributed to the writing and final approval of the manuscript.

## Conflict of Interest Statement

The authors declare that the research was conducted in the absence of any commercial or financial relationships that could be construed as a potential conflict of interest.
